# Rutin loaded bilosomes for enhancing the oral activity and nephroprotective effects of rutin in potassium dichromate induced acute nephrotoxicity in rats

**DOI:** 10.1038/s41598-024-73567-6

**Published:** 2024-10-11

**Authors:** Amira Mohamed Mohsen, Marwa Anwar Wagdi, Abeer Salama

**Affiliations:** 1 Pharmaceutical Technology Department, National Research Centre, El-Buhouth St., Dokki, Cairo, 12622 Egypt; 2 Pharmacology Department, National Research Centre, El-Buhouth St., Dokki, Cairo, 12622 Egypt

**Keywords:** Bilosomes, Rutin, Acute renal injury; Potassium dichromate; Akt/PI3K, Drug delivery, Kidney diseases

## Abstract

Rutin, a flavone glycoside, has shown to have a significant beneficial kidney protection effect in drug-induced nephropathy. However, its poor solubility and low oral bioavailability have limited its pharmacological applications. This study aimed at formulating rutin-loaded bilosomes to enhance the renal protective effect of rutin for oral application. Rutin-loaded bilosomes were developed using thin-film hydration technique. The prepared formulations were characterized by entrapment efficiency percentage (EE%), vesicular size (VS) and zeta potential (ZP) measurement. The developed formula exhibited moderate EE%, ranging from 20.02 ± 2.85 to 48.57 ± 3.57%, suitable VS results that ranged from 502.1 ± 36 to 665.1 ± 45 nm and high ZP values (≤ -41.4 ± 7.27 mV). Transmission electron microscopy revealed the spherical shape of the developed bilosomes. The *in-vitro* release study revealed prolonged release of rutin from bilosomes, relative to free drug. F_2_, prepared using the molar ratio span 60: cholesterol: sodium cholate 1:1:0.5, was selected for further investigations as it showed the highest EE%, smallest VS, optimum ZP, and persistent release profile. *In-vivo* studies were performed on drug-induced nephropathy in rats. Acute renal failure was induced using a single dose of potassium dichromate (PDC; 15 mg/kg; i.p). The selected formulation, F2, alleviated kidney dysfunction, oxidative stress and inflammation via decreasing MDA, TNF-α and TGF-β and increasing GSH. In addition, F2 promoted Akt/PI3K activation against PDC-induced acute renal failure. Histopathology results came in accordance with *in-vivo* results. Thus, bilosomes could be considered a potential delivery system for enhancing the oral delivery and kidney protection activity of rutin.

## Introduction

Acute kidney injury is a serious renal disease characterized by a rapid reduction in kidney function. It is also a major risk factor for chronic kidney disease, resulting in 2 million deaths globally every year. Unfortunately, there is no effective drug therapy. Therefore, searching for new potency compounds with minimal side effects is still a major challenge^1^. Rutin (3-rhamnosyl-glucosylquercetin)^2^, a flavone glycoside found in many plant species as Polygonaceae and Rutaceae, is a drug with strong anti-inflammatory, anti-oxidant, antibacterial and immunomodulatory properties; exhibiting protective effects on the heart, the kidneys and the liver^3^. Moreover, many studies proved that rutin has significant beneficial renal protective effect in drug-induced nephropathy, diabetic nephropathy and ischemia/reperfusion renal injury^4^. Rutin has the ability to scavenge ROS (reactive oxygen species) by donating hydrogen atoms to hydroxyl radicals, singlet oxygen, superoxide anions and peroxy radicals; furthermore rutin possess metal-chelating ability, which prevent metal ion-induced peroxidation^5^. Therefore, rutin has the ability to reverse oxidative stress which is essential in the nephrotoxicity process^6^. Unfortunately, the pharmacological application of rutin as an orally administered drug is restricted because of its poor solubility (0.125 g/L) and low oral bioavailability^3,7^.

Nanotechnology has made it possible for researchers to look deeply into more potent therapies. Many efforts have been made to increase the medications’ efficacy and safety by integrating them into different nanosystems^8^. In the efforts to enhance oral bioavailability of rutin, different nanocarriers have been employed to improve its therapeutic efficacy and its storage stability^9^. Rutin have been formulated in liposomes^10,11^, PLGA nanoparticles^12^, solid lipid nanoparticles^13^, inclusion complexes^14^ and phyto-sterosomes^15^. Recently, vesicular carriers have been considered as potential drug delivery systems^16^. Bilosomes (bile salts stabilized nanovesicles), are emerging as a new form of vesicular carriers for enhancing the oral delivery of encapsulated drugs. They have been reported to provide enhanced therapeutic efficacy to many natural compounds via different routes^17–22^. Bilosomes were initially described in 2001 by Conacher et al. in their pioneering works^23^ with more studies being carried out over the last decade proving that bilosomes have the ability to enhance in-vivo drug performance following oral administration^24^.

Bilosomes are closed bilayer nanovesicles of non-ionic amphiphiles similar to niosomes but embodying bile salts^25^. They are frequently prepared with non-ionic surfactants, such as span series, in addition to cholesterol and bile salts. The incorporation of bile salts into bilosomes offers better gastrointestinal stability in comparison to conventional niosomes. Bile salts also function as edge activators which destabilize the bilayer by reducing its surface tension, resulting in deformable vesicles with improved tissue penetration, triggering superior oral drug bioavailability after bilosomal encapsulation^24,26^. Cholesterol improve the rigidity of the bilayers and thereby increase drug entrapment^27^. These components used in the preparation of bilosomes are generally regarded as safe and have been proved to be biodegradable and biocompatible^24^.

The aim of the present work was to develop novel rutin-loaded bilosomes as an effective oral delivery system to enhance oral activity and renal protective effects of rutin in potassium dichromate-induced acute renal injury in rats.

## Materials and methods

### Materials

#### Chemicals

Rutin was supplied as generous gift sample from Kahira Pharmaceuticals and Chemical industries Co. (Cairo, Egypt). Sorbitan monostearate (Span 60), sorbitan monopalmitate (Span 40), methanol HPLC, cellulose membrane (molecular weight cut-off 12,000–14,000 g/mole), potassium dichromate (PDC) and cholesterol from Lanolin, minimum 99% (GC) were provided from Sigma-Aldrich, St. Louis, MO, USA. Sodium cholate 99% (SC) was procured from Acros Organics, Belgium. Chloroform HPLC was provided by Fisher Scientific, UK. Tween 80 was bought from Loba Chemie Co., India. Malondialdehyde (MDA) and reduced glutathione (GSH) kits were bought from Biodiagnostic kits® (Egypt). Phosphoinositide 3-kinase (PI3K), transforming growth factor-beta (TGF-β), tumor necrosis factor-alpha (TNF-α) as well as protein kinase B (Akt) ELISA kits were purchased from Sunlong Biotech Co., LTD, China. Other chemicals or solvents were of analytical grade.

#### Animals

Wister albino male rats (140–150 g) were supplied by the Animal House of the National Research Centre (Cairo, Egypt). Animals were kept in groups at controlled temperature and light (24 ± 2 ℃, 12/12-h light/dark cycle) with free access to water and standard laboratory rat chow. *In-vivo* studies were carried out according to the Animal Research Reporting of In Vivo Experiments (ARRIVE) guidelines and complied with the National Institutes of Health guide for the care and use of Laboratory animals (NIH Publications No. 8023, revised 1978). The institutional ethical committee [Medical Research Ethics Committee (MREC), NRC] approved the experimental protocol (Number 13020252).

### Methods

#### Preparation of rutin-loaded bilosomes

Rutin-loaded bilosomes were prepared adopting thin-film hydration method, stated by Baillie et al.^28^, commonly used for the preparation surfactant-based vesicles^29^. 100 mg of non-ionic surfactants (Span 60 or Span 40) and cholesterol in a molar ratio of 1:1 or 2:1 together with 20 mg of rutin were dissolved in 10 ml chloroform: methanol mixture (2:1) in a long neck quick fit round-bottom flask which was fitted in a rotary evaporator (Büchi-M/hb-140, Switzerland). The organic solvent was evaporated under vacuum, at an adjusted temperature of 60 ± 0.5 ℃ and a constant rotation speed (150 rpm), till the formation of a thin dried film onto the flask wall. The film was hydrated with 10 mL phosphate buffer (pH 7.4), containing sodium cholate in 2 different concentrations, under rotation at 60 ± 0.5 ℃ for half an hour to obtain a bilosomal suspension^30^. The composition of rutin-loaded bilosomes was illustrated in Table 1.

**Table 1:** Composition and characterization parameters of the developed rutin loaded bilosomes.

Formulae code	Molar ratio				EE% ± S.D	Drug loading ± S.D. (%)	VS ± S.D. (nm)	PDI ± S.D	ZP ± S.D. (mV)
	Span 60	Span 40	Cholesterol	Sodium cholate					
F1	1	–	1	0.25	36.84 ± 4.56	3.55 ± 0.31	646.6 ± 41	0.410 ± 0.025	− 44.7 ± 7.84
F2	1	–	1	0.5	48.57 ± 3.57	4.63 ± 0.27	502.1 ± 36	0.351 ± 0.057	− 47.3 ± 6.16
F3	–	1	1	0.25	21.07 ± 3.92	2.06 ± 0.13	665.1 ± 45	0.456 ± 0.069	− 45.9 ± 9.18
F4	–	1	1	0.5	28.57 ± 3.14	2.77 ± 0.24	513.2 ± 39	0.412 ± 0.045	− 50 ± 7.37
F5	2	–	1	0.25	23.92 ± 3.82	2.33 ± 0.19	603 ± 32	0.391 ± 0.055	− 41.4 ± 7.27
F6	2	–	1	0.5	47.14 ± 0.71	4.50 ± 0.31	522 ± ± 29	0.341 ± 0.039	− 49 ± 10.5
F7	–	2	1	0.25	20.02 ± 2.85	1.96 ± 0.15	652 ± 35	0.490 ± 0.068	− 47.3 ± 9.54
F8	–	2	1	0.5	22.14 ± 4.28	2.16 ± 0.28	566.3 ± 31	0.431 ± 0.074	− 49.1 ± 10.9

#### Characterization of rutin-loaded bilosomes

##### Entrapment efficiency (EE %)

Non-entrapped rutin was separated from bilosomal suspensions by cooling centrifugation (Union 32R, Korea) rotating at 7000 rpm (at 5200 × g), at − 4 ℃ for one hour. Supernatants were collected and analysed for free rutin spectrophotometrically at λ_max_ 257 nm utilizing UV- Visible spectrophotometer (Shimadzu UV Spectrophotometer (2401/PC), Japan)^31,32^, after comparison to a pre-constructed calibration curve (n = 3, R^2^ = 0.999). The regression equation of standard curve was y = 0.035x + 0.0012.

Entrapment efficiency (%) and Drug Loading (%) were estimated as follows^27^:

$$\text{EE \%}=\frac{\text{Total amount of drug - Amount of free drug}}{\text{Total amount of drug }}   \times 100,$$

$$\text{DL\%}=\frac{\text{ED}}{(\text{Amount of Span}+\text{Amount of SC})} \times 100,$$

where ED is the amount of entrapped drug and SC is sodium cholate.

##### Vesicle size (VS), polydispersity index (PDI) and zeta-potential determination (ZP)

Zeta sizer (Malvern Zeta-sizer Nano ZS, Malvern Instruments, UK) was utilized to estimate VS, ZP and PDI of all prepared bilosomes. 0.1 ml of each formula was diluted by 10 ml bidistilled water^33^, then the sample was put in a quartz cuvette and examined at 25 ℃^34^.

##### Transmission electron microscopy (TEM)

The morphological aspect of F_2_ and F6 was studied using TEM (JEOL, JEM-1230, Tokyo, Japan) after suitable dilution. A drop was placed upon the carbon-coated grid 300-mesh and left for 15 min to be air-dried at room temperature. One droplet of a negative staining agent (1% phosphotungestic acid solution) was added, the excess was removed using filter paper. Specimens were left for 2 min at room temperature to dry in order to be examined using TEM at 25 ± 0.5 ℃, then micrographs were recorded at appropriate magnifications^2^.

##### Fourier transform infrared spectroscopy (FT-IR)

Rutin, Span60, Cholesterol, SC, plain bilosomes and rutin-loaded bilosomes were analyzed using FT-IR spectrophotometer (JASCO 6100, Tokyo, Japan). Each sample was combined separately with potassium bromide and compacted under 200 kg/cm^2^ of hydraulic pressure for two minutes to create compact disks. Every sample was scanned using a frequency range of 4000 to 400 cm^−1^, all against a background of blank KBr pellets^35^.

#### *In-vitro* release study

##### *In-vitro* release profiles

*In-vitro* release of rutin from selected bilosomes was studied utilizing dialysis membrane diffusion technique in the shaking water bath (Memmert, SV 1422, Schwabach, Germany)^36^. An amount, equivalent to 2 mg rutin, was withdrawn from the selected formulations and from free rutin suspension and transferred into previously hydrated dialysis bags (Spectra/Por® having molecular weight cut off 12000–14000 Da) which were sealed at both sides. Dialysis bags were immersed into 100 ml phosphate buffer (pH 7.4) containing Tween 80 (0.5% w/v) to preserve sink conditions for the drug^2,37^ and rotated at 100 rpm under a well maintained temperature of 37 ± 0.5 ℃^2,13,38^. At fixed time intervals (1, 2, 3, 4, 5, 6, 8 and 24 h.), samples were taken, while an equal volume of fresh medium was added to maintain sink condition. Drug concentration was determined in each sample spectrophotometrically. Cumulative release percentage (ratio of the amount of rutin released to the initial amount of rutin) was computed at each time interval. Results were presented as mean of three replicates ± S.D. Cumulative release percentages were plotted vs. time^33^.

##### Drug release kinetics

Data of* in-vitro* release study were kinetically treated to predict the mechanism of rutin release from bilosomes. Drug release profiles were fitted with zero-order, first-order, second-order, Higuchi and Korsmeyer-Peppas equation. Release model having highest regression coefficient (R^2^) values was considered as appropriate fit model^30^.

#### *In-vivo* study

##### Study design

Rats were assigned in five groups (n = 8) at random. Acute renal failure was induced by intraperitoneal injection of a single dose of PDC (15 mg/kg)^39^. The first group (normal control) received single i.p. injection of saline and daily oral bidistilled water for 7 days. Group 2 received only PDC. Groups 3–5 received drug-free bilosomes (blank), free rutin, and the selected rutin-loaded bilosomes (F_2_), respectively, at a dose of 200 mg/kg orally^40^, for 7 days before PDC injection.

##### Serum biochemical analysis

In the end of the study, blood specimens were withdrawn from the retro-orbital vein of each animal in all groups after being anesthetized by intraperitoneal injection of sodium pentobarbital (40 mg/kg)^39^. Specimens were left 10 min at room temperature in order to stand, and then centrifuged in a cooling centrifuge (Laborezentrifugen, 2k15; Sigma, Germany) at 3000 rpm and 4 ℃ for 10 min. Serum creatinine and urea were estimated in the resultant serum samples.

##### Tissue biochemical analysis

Animals were sacrificed by cervical dislocation under anesthesia (sodium pentobarbital 40 mg/kg, i.p.), just after blood sampling^39^. Rats’ kidneys were dissected out and washed with phosphate buffer saline to remove residual blood. Right kidneys were homogenized (MPW-120 homogenizer, Med instruments, Poland). 20% homogenate was obtained, stored overnight at −20 ℃, then centrifuged using cooling centrifuge rotated at 5000 g for 5 min. Collected supernatants were stored at − 80 ℃ to be used for determination of MDA, GSH, TGF-β, TNF-α, Akt and PI3K contents in renal tissues using specific ELISA kits.

##### Histopathological examination

The left Kidneys were collected and fixed in 10% phosphate buffered formalin just after animals scarification. Renal tissue specimens were dehydrated then embedded into paraffin wax. Serial paraffin Sects. (4 µm) were obtained and left for more than twelve hours at 37 ℃. Sections were stained with haematoxylin and eosin (H&E) for examination using an optical microscope to study histopathological changes.

### Statistical analysis

Values were presented as means ± SD. Results of EE%, PS and ZP of investigated groups were compared utilizing one-way analysis of variance (ANOVA) followed by Fisher’s LSD test using SPSS software 17.0. Analyses of *in-vivo* data were carried out utilizing GraphPad Prism software, version 5 (GraphPad Prism Inc., USA). Differences were significant when p < 0.05.

## Results and discussion

### Preparation of rutin-loaded bilosomes

Drug loaded bilosomes were successfully developed employing thin film hydration method^41^. The two non-ionic surfactants (NIS) employed were Span 40 and Span 60 while one bile salt (sodium cholate, SC) was included in bilosomal formulations. Four molar ratios were used, namely, NIS: Cholesterol: bile salt (1:1:0.25), (1:1:0.5), (2:1:0.25) and (2:1:0.5). The composition of the developed formulae is represented in Table 1.

### Characterization of rutin-loaded bilosomes

#### Entrapment efficiency (EE%)

The EE% of rutin-loaded bilosomal formulae (Table 1) shows that all developed formulations exhibited good capability to encapsulate rutin, showing an EE% ranging from 20.02 ± 2.85 to 48.57 ± 3.57% and a DL% ranging from 1.96 ± 0.15 to 4.63 ± 0.27.The increase in the ratio of SC from 0.25 to 0.5 led to an increase in the EE% of rutin, where a significant (p < 0.05) increase was shown between F1 and F2, F3 and F4, F5 and F6, while a non-significant increase (p > 0.05) was observed between F7 and F8. This could be due to the surfactant characteristics of bile salts, which might integrate vertically into the surface of the bilayer membrane, disturb the lipid matrix’s a Vcyl chains, and enhance the membrane’s flexibility. This would lead to an enhancement of the solubility of lipophilic drugs in the membrane^42–44^ and hence enhancement of the drug entrapment.

The results also reveal that bilosomes developed with Span 60 had a higher EE% than those prepared with Span 40 (Table 1). There are three possible explanations for this. Firstly, the longer saturated alkyl chain (C18) of Span 60 results in a more stable bilosome bilayer^45,46^. Additionally, with a lower HLB value (4.7) compared to that of Span 40 (HLB 6.7), Span 60 is more hydrophobic, leading to improved EE%^47^%. Lastly, its solid consistency at room temperature and higher phase transition temperature (53 ℃) could aid in its stability by decreasing vesicles permeability in comparison with vesicles prepared using Span 40 having lower phase transition temperature (42 ℃)^46,48^.

#### Vesicle size (VS), Polydispersity index (PDI) and Zeta Potential (ZP)

Particle dimensions applied in nanoparticle delivery can vary between 10 and 1000 nm. All the prepared bilosomal formulae were in the nanometric dimensions (from 502.1 ± 36 to 665.1 ± 45 nm) (Table 1). Results showed that increasing the concentration of SC led to a significant (p < 0.05) decrease in vesicle size. This could be due to the enhanced flexibility and lower surface tension of the vesicles after increasing the amount of bile salt^49^, in addition to higher negative charge of the external layer. The results also showed the impact of type of surfactant, where bilosomes prepared using span 40 exhibited larger VS than those developed using span 60. This might be attributed to the higher surfactant hydrophilicity of span 40 (HLB 6.7) compared to that of span 60 (HLB 4.7). Higher HLB results in higher water uptake resulting in increasing the bulk size of vesicles^41^. The results presented in Table 1, also revealed that the PDI value of the developed formulae ranged from 0.351 to 0.490 (i.e. PDI values < 0.5), indicating a narrow size distribution with good homogeneity^50,51^.

The Zeta potential (ZP) values of the bilosomal vesicles assessed in this research were highly negatively charged, ranging from − 41.4 ± 7.27 to − 50.1 ± 7.37 mV, indicating outstanding physical stability for all the created systems. Heightened ZP values (greater than 30 mV) signify electrostatic repulsion among charged particles, preventing particle clumping and thereby boosting physical stability^52^. The negative ZP is likely due to the presence of Span 40 or Span 60. At a neutral pH, nanostructured lipid carriers generate a negatively charged surface due to the selective absorption of hydroxyl ions at the vesicle’s surface^53,54^. Moreover, incorporating negatively charged bile salts increased the negative charge. It was noted that increasing the bile salts amount led to a rise in the absolute ZP value. Statistical analysis revealed that formulae prepared using higher amounts of SC (F2, F4, F6 and F8) acquired significantly higher absolute ZP values than their corresponding formulations prepared using lower amounts of bile salt (F1, F3, F5 and F7) (p < 0.05). ZP values came in accordance with VS results, where ZP of span 40 vesicles (F3, F4, F7 and F8) was significantly higher than span 60 bilosomes (F1, F2, F5 and F6) (p < 0.05). Higher ZP values enhances the repulsion force between bilosomal bilayers resulting in an increase in the VS^55^.

**Fig. 1:**
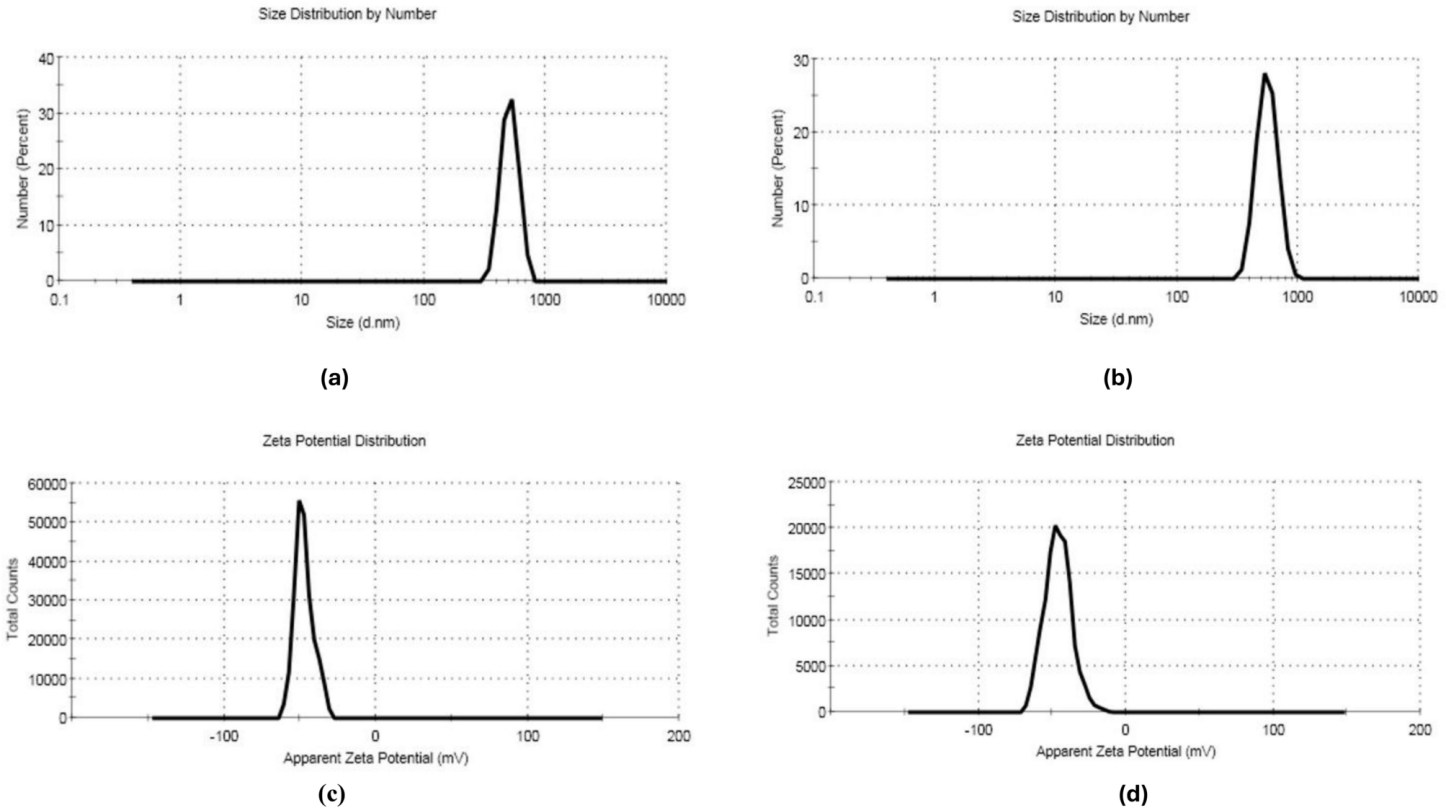
PS and ZP graphs of selected rutin-loaded bilosomes F2 (**a, c**) and F6 (**b, d**).

Based on the abovementioned results, the bilosomal formulations F2 and F6 revealed the highest EE%, as well as the small vesicle size and excellent physical stability (high ZP values). Thus, they were chosen for further studies. The graphs of particle size and zeta potential of F2 and F6 are demonstrated in Fig. 1.

#### Transmission electron microscopy

**Fig. 2:**
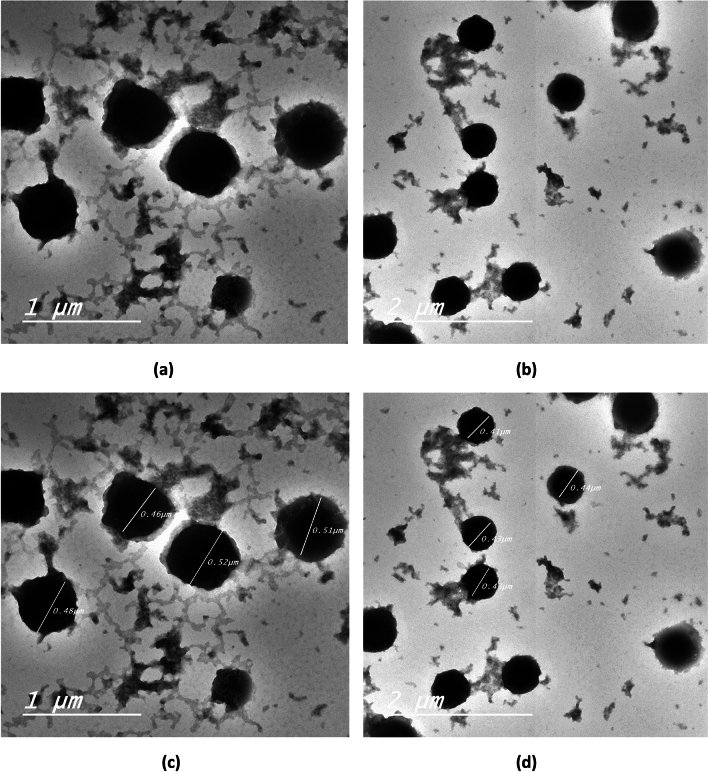
TEM micrographs of selected rutin-loaded bilosomes; F2 (**a, c**) and F6 (**b, d**).

TEM micrographs of rutin-loaded bilosomes F2 and F6 are demonstrated in Fig. 2. Clear outlines of spherical-shaped vesicles were visible in both micrographs. The vesicles were well identified and homogenously dispersed. TEM-obtained vesicle sizes were marginally smaller than the vesicle size analysis results. This might be because Zetasizer can measure the hydrodynamic layer around the vesicle, whereas TEM can only see the vesicle^56^.

#### Fourier transforms infrared (FT-IR) spectroscopy

**Fig. 3:**
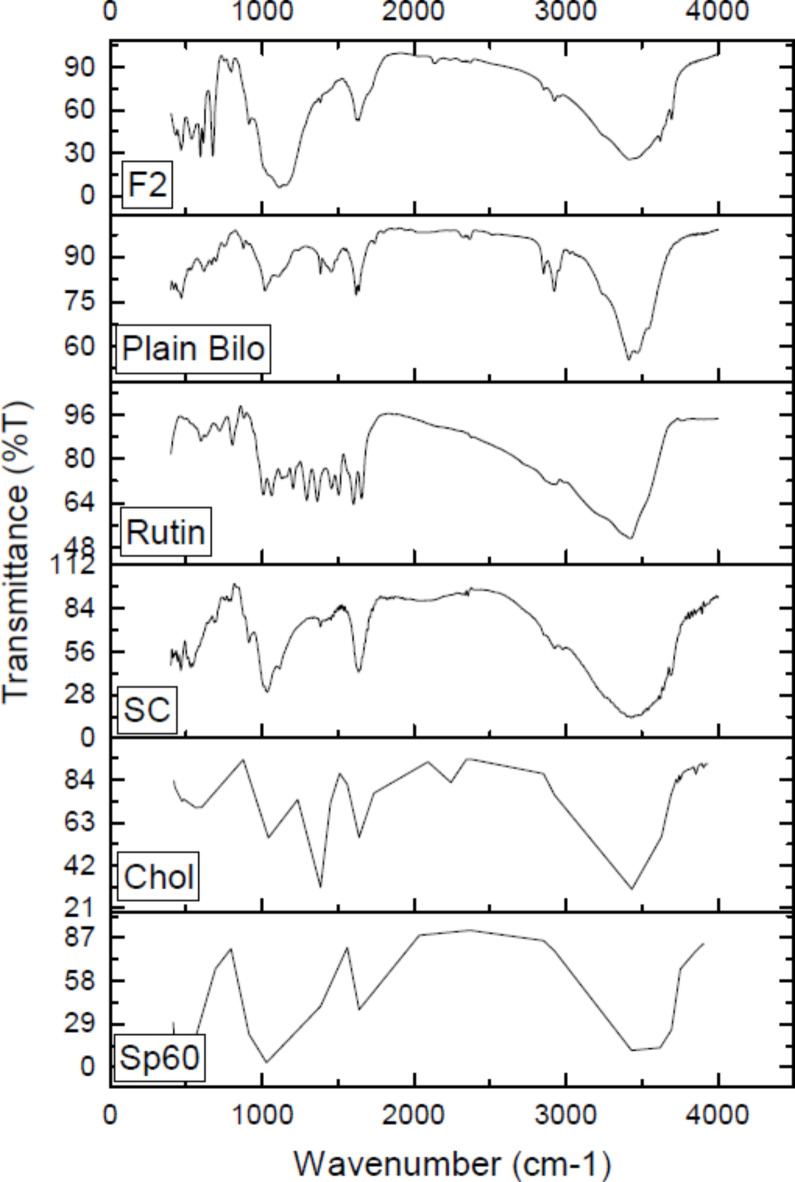
FT-IR spectra of rutin, plain bilosomes, rutin loaded bilosomes (F2) and individual components of bilosomes.

Figure 3 reveals the FT-IR spectra for rutin, plain bilosomes, rutin loaded bilosomes in addition to the individual components of bilosomal formulation (Span60, Cholesterol, SC). FT-IR spectrum of Span 60 showed peaks for the strong C = O ester bond at 1734.8 cm^−1^, the hydroxyl group at 3377.5 cm^−1^, and the asymmetric and symmetric aliphatic C-H stretching at 2916.3 & 2849.2 cm^−1^, respectively^57^. The distinctive peaks in the cholesterol spectrum corresponded to the hydroxy group and carboxylic acid functional group which were located at 3426.4 and 2930.5 cm^−1^, respectively^58^.

SC spectrum showed a broad band at 3488.59 cm^−1^ which is assigned for O–H bond stretching. The spectrum also revealed a sharp peak at 1635.34 cm^−1^ which is assigned for –C = O group of carboxylate ester group in SC. Another sharp peak of CCO bonds appeared at 1031.73 cm^−1^^59^ FT-IR spectrum of plain bilosomes revealed a broad peak at 3415.314 which might correspond to a shift of O–H bond stretching of SC. Sharp peaks appeared at 1619.91 and 1020.15 which corresponds to -C-O group and CCO groups, respectively, which are characteristic peaks of SC,. Moreover, another peak appeared at 2852.2026, which is a shift of C-H stretching of Span 60.

Rutin’s FT-IR spectrum showed its characteristic bands at 3423.03 cm^−1^ (OH bonded), 1655.59 cm^−1^ (C = O stretch) and 1601.59 cm^−1^ (aromatic structure). These peaks were shifted, in the rutin-loaded bilosomes spectrum, to 3417.24 cm^−1^ (OH bonded), 1635.34 cm^−1^ (C = O stretch) and 1621.84 cm^−1^. FT-IR spectrum of rutin-loaded bilosomes revealed also a peak at 1114.65 which might correspond to a shift of the characteristic peak of SC. FT-IR analysis of rutin-loaded bilosomes (F2) displayed the distinctive peaks of the drug and ingredients with a slight shifting, confirming the absence of any chemical interactions between the drug and the excipients. This comes in accordance with previously reported studies^60,61^.

### *In-vitro* release studies

#### *In-vitro *release profiles

**Fig. 4:**
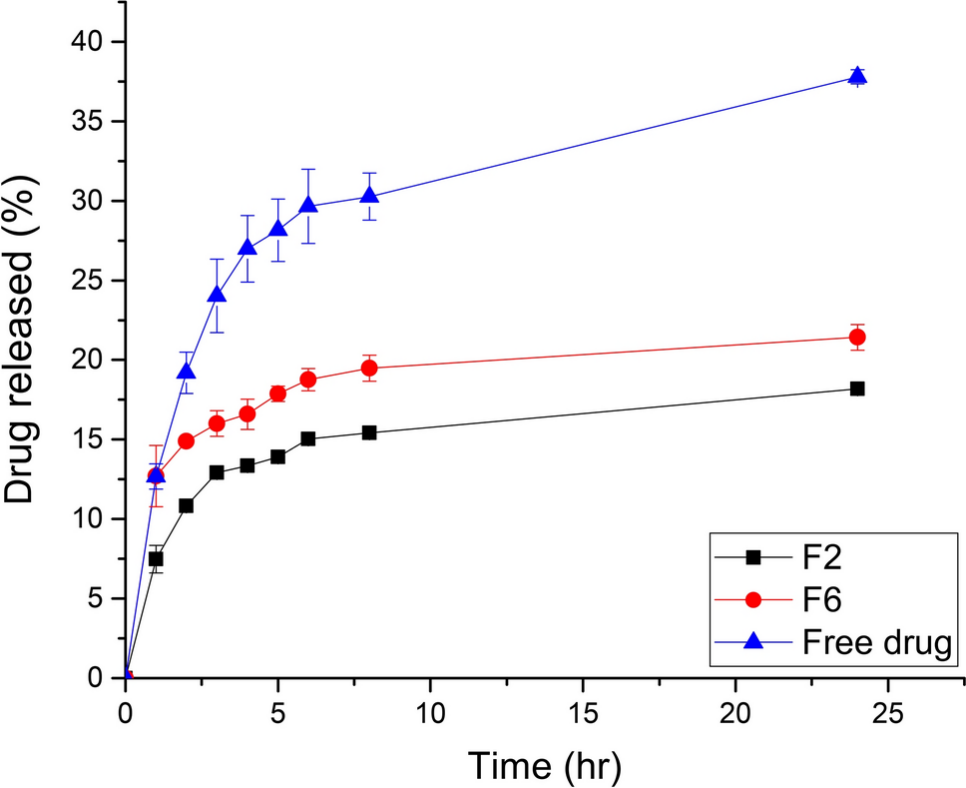
*In-vitro* release profiles of rutin from the selected bilosomes (F2 and F6) compared to free drug suspension at 37 ℃ (n = 3).

*In-vitro* release of drug from the bilosomal formulation as well as free drug suspension are shown in Fig. 4. The profiles reveal a biphasic release pattern, where the drug release exhibited a fast release pattern which was followed by a sustained release profile. The release of drug from bilosomal formulations showed more sustained release effect, compared to free drug suspension. The prolonged release of rutin from the formulations under investigation is attributed to the benefit of bilosomes as colloidal particle nanocarriers in comparison to traditional dosage forms. They have the potential to act as drug reservoirs, allowing the entrapped medication to release gradually^62^. Furthermore, it is possible to attribute the delayed release of rutin from the vesicular formulations to the lipophilic rutin’s high affinity for the hydrophobic moieties in the formulations. Additionally, by reducing the fluidity of the vesicular membrane, the use of cholesterol in the vesicle preparation process reduces the permeability or release of the drug that is entrapped.

#### Drug release kinetics

**Fig. 5:**
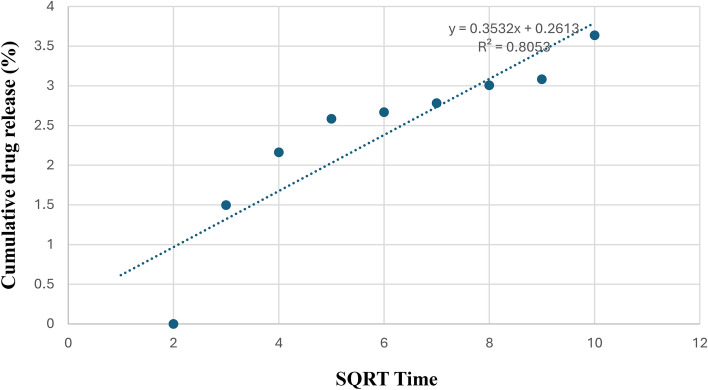
Higuchi release kinetic graph of optimized formulation F2.

Table 2 reveals the analysis of rutin release kinetics data from the prepared bilosomal formulae. After analyzing the *in-vitro* release data using various models, it was found to be best matched to Higuchi’s equation based on the highest correlation coefficients. A deeper understanding of the mechanism governing the release can be obtained by applying Peppas model analysis to the release data and by clarifying the release exponents “n”. Table 2 reports good linearity (R^2^ = 0.9016–0.9092). As previously reported by Peppas et. al., the drug release follows the Fickian diffusion if n ≤ 0.43, anomalous (non-Fickian) diffusion if 0.43 < n < 0.85, case II transport if n = 0.85 and super-case II transport if n > 0.85. According to Table 2, the release exponent “n” values were ≤ 0.43, suggesting a Fickian diffusion mechanism. This result is consistent with previously reported study^63^. F2, which showed the highest EE%, smallest VS, optimum ZP, and persistent release profile, was chosen for more investigations. The release kinetic graph of the optimized formulation F2 is presented in Fig. 5.

**Table 2:** The calculated correlation coefficients and kinetics parameters of rutin release profiles from the developed bilosomes.

Code	Zero order	First order	Higuchi	Peppas	
	R^2^			R^2^	n
F2	0.6306	0.4962	0.8053	0.9016	0.2032
F6	0.5634	0.5117	0.755	0.9092	0.2556

### *In-vivo* studies

#### Effect of rutin-loaded bilosomes on renal function

Acute kidney injury (AKI) is a clinical problem in which severe renal tubules damage with a sudden renal function decline is observed. PDC, a toxic form of chromium, provokes AKI through the production of ROS such as MDA and also mediates inflammatory process via TNF-α and TGF-β over expression^64^.

**Fig. 6:**
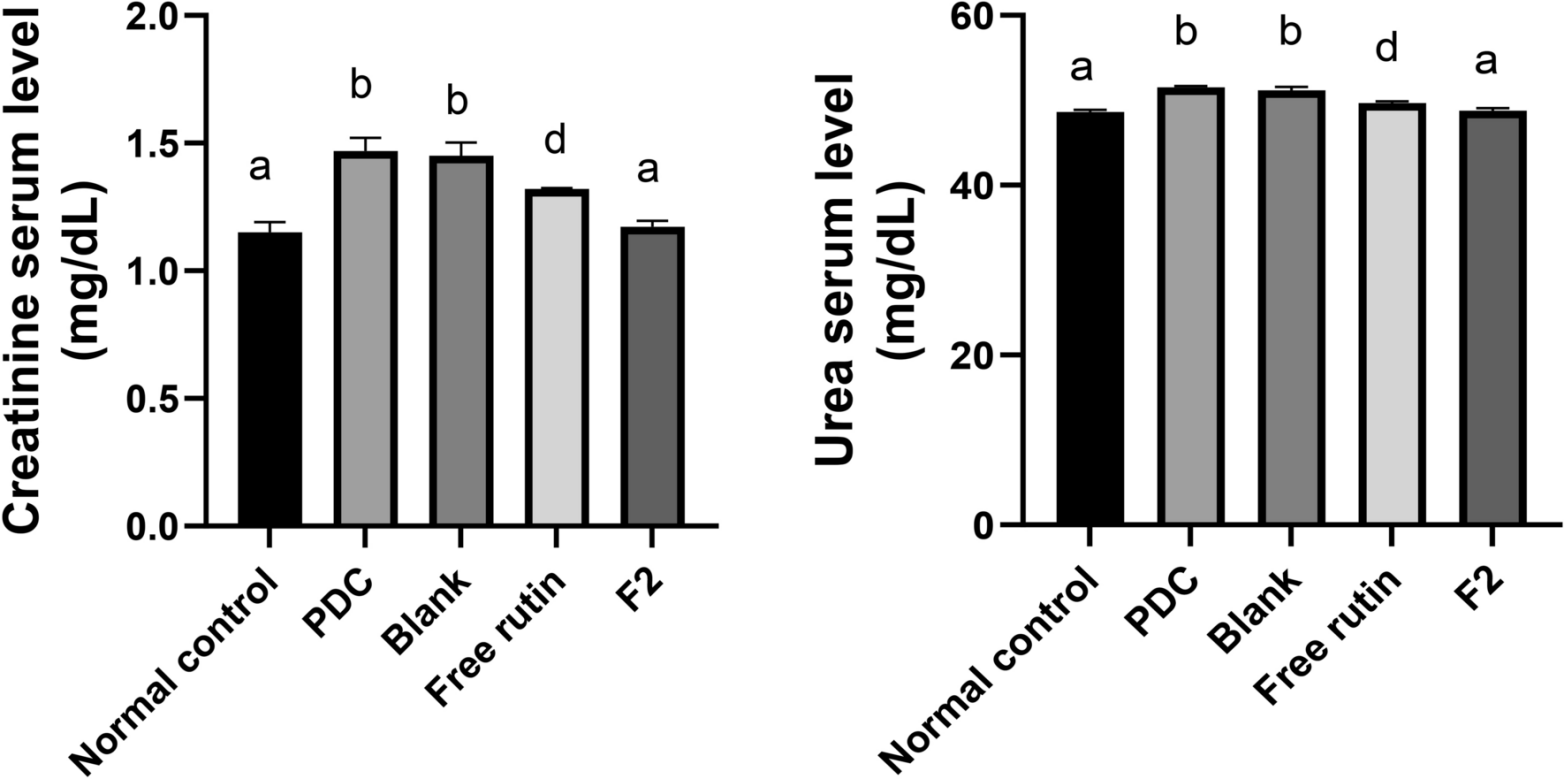
Effect of blank bilosomes, rutin-loaded bilosomes (F2) and free rutin on renal function. All the values are expressed as means ± standard deviation of the means (SD). Different groups comparisons were carried out using one-way analysis of variance (ANOVA) followed by Fisher’s LSD test. Same letter means non-significant difference, while different letter means significant difference at p < 0.05.

Our results revealed that the renal function parameters were significantly (p < 0.05) elevated in PDC rats; creatinine and urea serum levels were elevated by 28 and 6% respectively, as compared to normal control. The administration of free rutin & F_2_ significantly (p < 0.05). Reduced serum levels of creatinine by 10 and 20%; and urea by 4 and 5% respectively, as compared to PDC group. Furthermore, F2 significantly (p < 0.05) enhanced the elevation in creatinine and urea by 19 and 5% as compared to free rutin. It was obvious that treatment with F_2_ returned creatinine and urea levels to normal values as there is no significant difference between the normal control and F_2_ groups regarding creatinine and urea levels (p > 0.05) (Fig. 6). Previously, rutin protected against kidney disease induced by adenine through improving kidney function^65^.

#### Effect of rutin-loaded bilosomes on oxidative stress and AKT/PI3K pathway in renal tissue

**Fig. 7:**
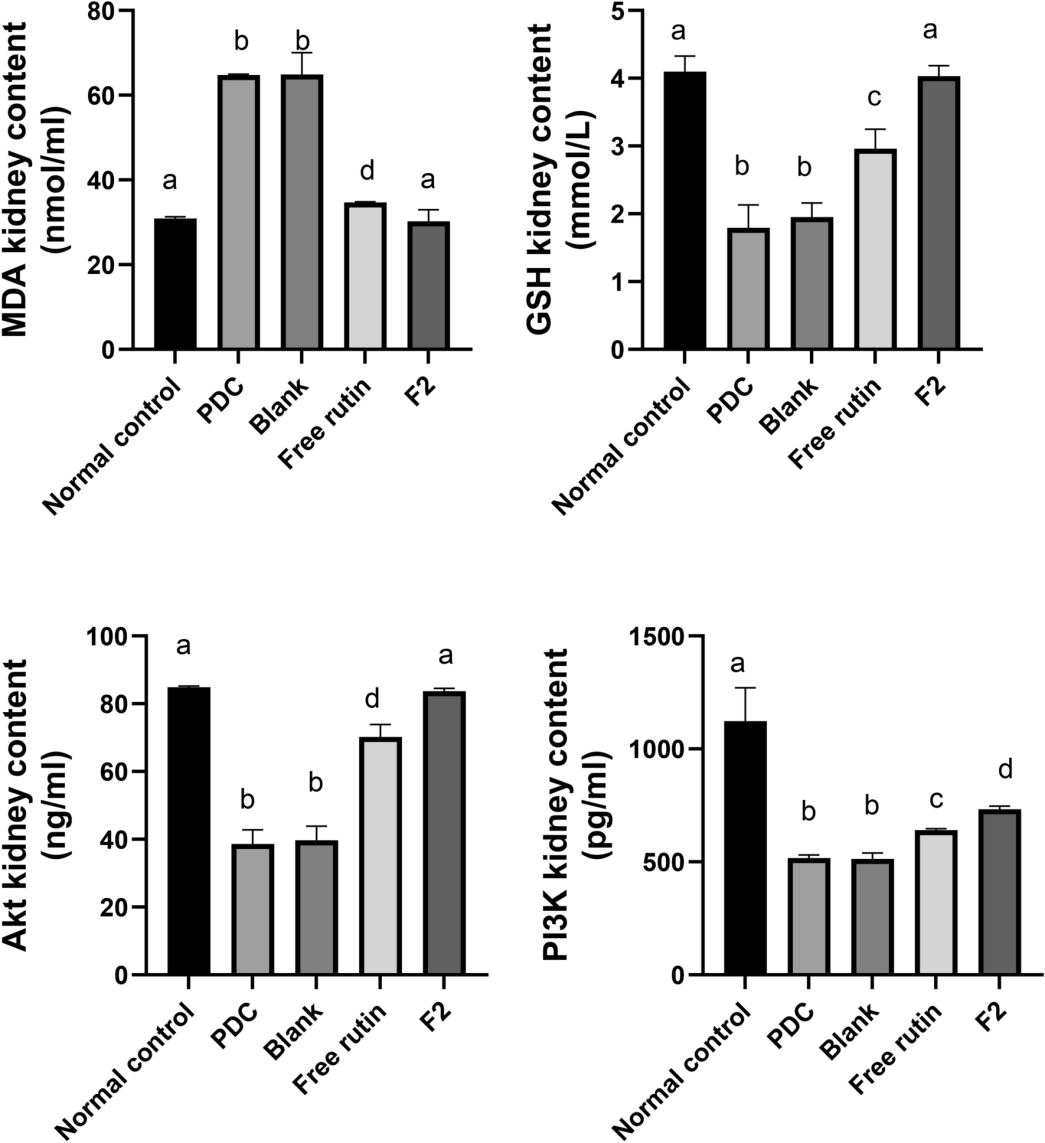
Effect of blank bilosomes, rutin-loaded bilosomes (F2) and free rutin on oxidative stress and AKT/PI3K pathway in renal tissue. All the values are expressed as means ± standard deviation of the means (SD). Different groups comparisons were carried out using one-way analysis of variance (ANOVA) followed by Fisher’s LSD test. Same letter means non-significant difference, while different letter means significant difference at p < 0.05.

There is a significant reduction in the renal content of GSH by 56% in the PDC group with an elevation of MDA renal content by 109%, compared to the normal rats (p < 0.05). Treatment with free rutin & F_2_ resulted in a significant (p < 0.05). Elevation of the renal content of GSH by 65 and 125%, respectively, however free rutin & F2 treatments resulted in a significant (p < 0.05) reduction in renal MDA level by 46 and 53%, respectively, compared to the PDC group. Moreover, F2 treatment was superior to free rutin by causing a significant elevation (p < 0.05) in GSH by 107% as well as a significant decrease (p < 0.05) in MDA by 53%. Furthermore, F2 treatment returned the GSH and MDA level to their normal value as there is non-significant difference between F_2_ and normal groups in the levels of GSH and MDA (p > 0.05) (Fig. 7). Rutin nanoparticles, in previous study, upregulated Nrf-2/HO-1/ GSH antioxidant pathway and decreased MDA ameliorating diabetic nephropathy^66^.

PDC down-regulates Akt/PI3K gene in kidney tissues, inducing apoptosis. The Akt/PI3K pathway plays a crucial role in protein synthesis and cellular defense against oxidative damage. PI3K activates Akt which in turn enhances cellular growth and autophagy^39^. Analysis of our data revealed a significant reduction (p < 0.05) of Akt and PI3K in PDC rats by 55 and 54% respectively, as compared to normal rats. Treatment with free rutin & F_2_ resulted in a significant (p < 0.05) enhancement of the renal content of Akt by 82 and 117%, respectively, also, free rutin & F_2_ resulted in a significant (p < 0.05) increase of renal PI3K level by 24 and 42%, respectively, compared to the PDC group. Moreover, F2 treatment resulted in a significant elevation (p < 0.05) of Akt/PI3K kidney contents by 111 and 43% as compared to free rutin and returned Akt to its normal value where there is a non-significant difference (p > 0.05) in Akt level between F2 and normal groups (Fig. 7). These results suggest kidney cryoprotection of rutin, for the first time, via up regulating Akt/PI3K signaling pathway. Previously, another flavonoid apigenin stimulated Akt and contributed to protect human renal proximal tubular epithelial (HK-2) cells preventing cisplatin cytotoxicity^67^.

#### Effect of rutin-loaded bilosomes on inflammatory mediators

**Fig. 8:**
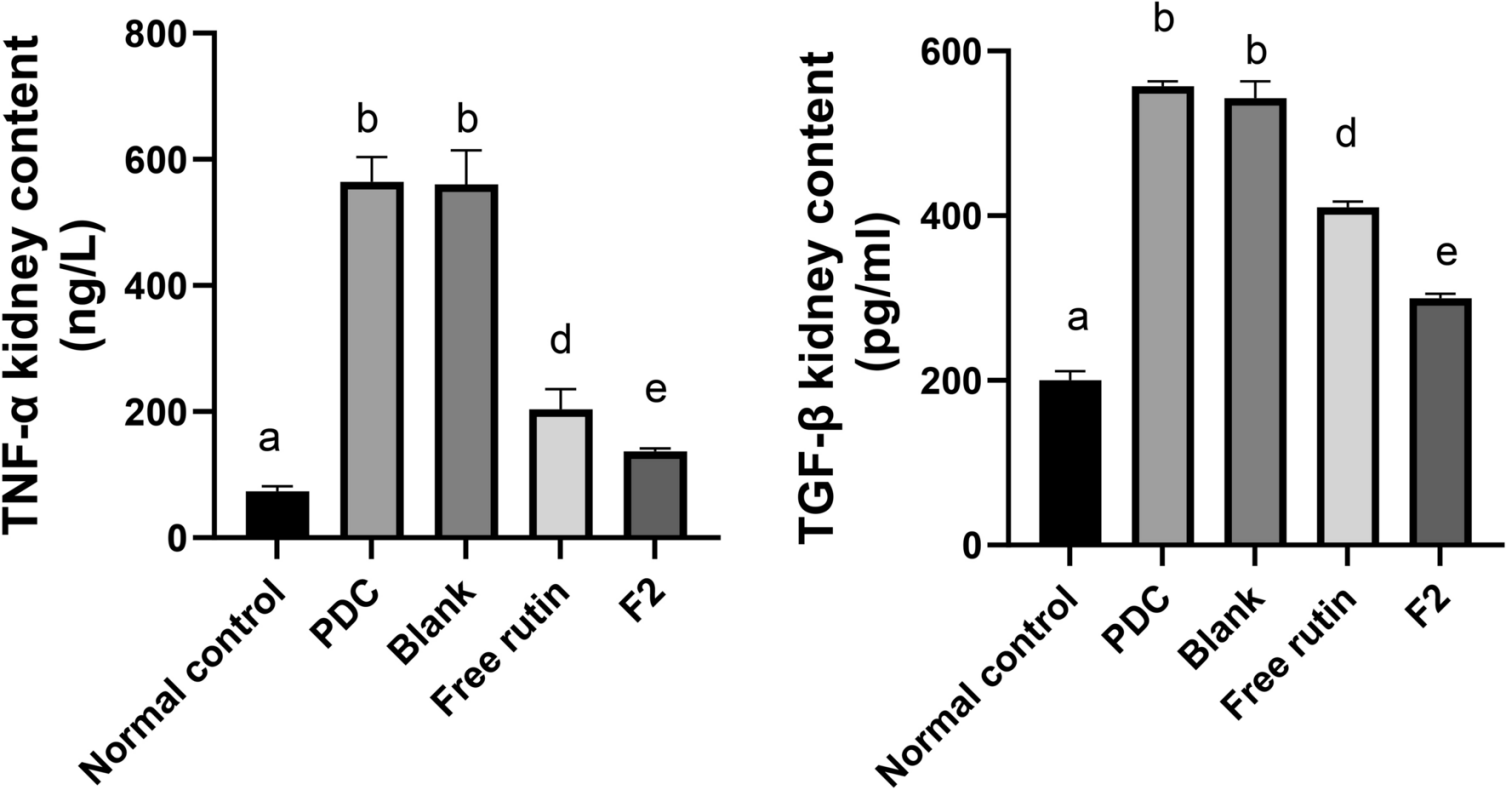
Effect of blank bilosomes, rutin-loaded bilosomes (F2) and free rutin on inflammatory mediators. All the values are expressed as means ± standard deviation of the means (SD). Different groups comparisons were carried out using one-way analysis of variance (ANOVA) followed by Fisher’s LSD test. Same letter means non-significant difference, while different letter means significant difference at p < 0.05.

Renal cells expressed TNF-α proinflammatory cytokines and mediated inflammatory response and inducing AKI^68^. PDC, in this study, induced the release of inflammatory mediators like TNF-α and TGF-β by 6.6-fold and 1.7 folds, respectively, as compared with normal control group. The use of free rutin & F_2_ treatments significantly decreased (p < 0.05) TNF-α renal content by 64 and 76% as well as TGF-β renal content by 26 and 46%, respectively, as compared to PDC group. Moreover, F2 significantly reduced (p < 0.05) TNF-α and TGF-β kidney contents by 76 and 45% as compared to free rutin (Fig. 8). Flavonoids have anti-inflammatory effects due to suppression of IL-6, TNF-α, TGF-β1 in nephritis^69^.

#### Histopathological studies

**Fig. 9:**
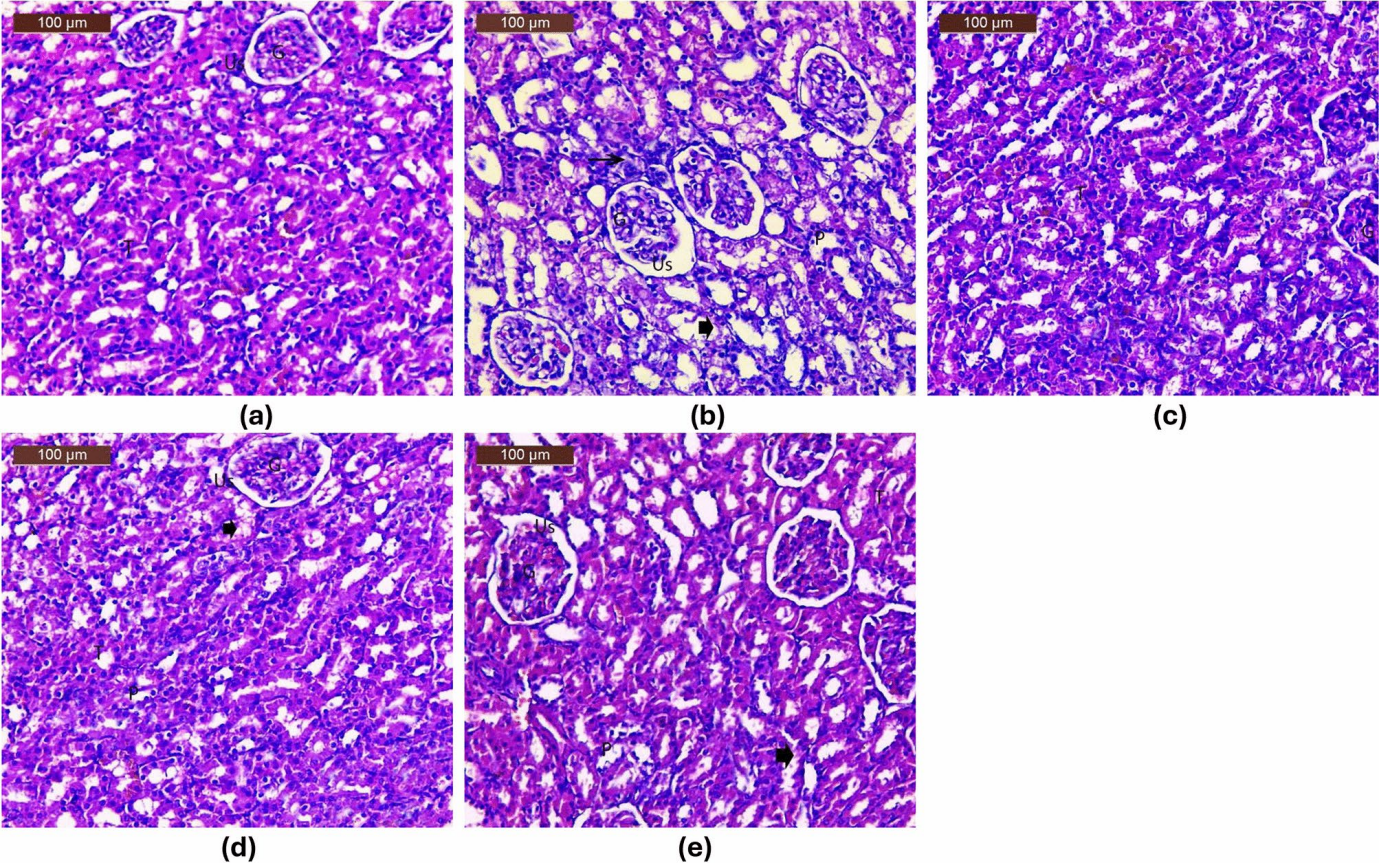
Photomicrographs of H & E-stained histological sections of normal, Potassium dichromate (PDC)-intoxicated, standard and test drug treated rats’ kidney. (**a**) Normal control; (**b**) PDC (positive control); (**c**) PDC + blank niosomes; (**d**) PDC + F2 bilosomes, (**e**) PDC + free rutin, where glomerulus is symbolized as (G); urinary space (Us), tubules (T); tubular epithelial cell degeneration (arrowhead); pyknotic nuclei (P); mild inflammatory cells (arrow).

Sections of the kidney from the control group displayed normal tubule architecture (T), normal urine space (Us), and normal Bowman’s space between the glomeruli (G) (Fig. 9a). Sections of rat kidneys treated with PDC were examined histopathologically, and the results revealed mildly inflammatory cells (arrow), tubular epithelial cell degeneration (arrowhead) with present pyknotic cells (P), and shrunken glomeruli (G) with an unusually wide urine space (US) (Fig. 9b). The kidneys of animals treated with PDC and blank (drug-free) bilosome exhibited minimal ameliorative effects, including slight interstitial bleeding, dilated urinary space (Us), mitigated tubular degeneration (arrowhead), and atrophy of glomeruli (G) (Fig. 9c).

Kidneys of animals treated with PDC, and free drug showed moderately ameliorative effect with mild pyknotic nuclei (P), mild tubular degeneration (arrowhead), mild dilated urine space (Us), and slight atrophy of glomeruli (G) (Fig. 9d). Kidneys from animals treated with PDC and rutin-loaded bilosomes F2 showed relatively normal histological structure; where renal corpuscles were apparently normal and were formed of a rounded glomerulus (G), with relatively normal urinary space (Us) in-between. Tubular epithelial apparently nearly normal with few tubules exhibit pyknotic nuclei (P) (Fig. 9e).

In conclusion, the biological evaluation results revealed that the kidney protection effect of rutin in drug-induced nephropathy was significantly improved via loading into bilosomes. This could be attributed to the enhancement of intestinal membrane permeability due to the ability of the incorporated bile salts to fluidize membrane lipid bilayer with subsequent improvement of intestinal permeation and to the solubilizing effect of Span 60 which act as a penetration enhancer^24,70^. Moreover, the integration of bile salts in the bilayer make vesicles resistant to physiological bile salts in the GIT^70^. These results come in accordance with previous studies which suggested that bilosomes might serve as potential carriers to enhance the oral bioavailability of drugs^33,36^.

## Conclusion

The developed rutin-loaded bilosomes exhibited relatively moderate entrapment efficiency percentage (20.02 ± 2.85–48.57 ± 3.57%), suitable vesicle size (502.1 ± 36–665.1 ± 45 nm) and high zeta potential values ≤  − 41.4 ± 7.27. Transmission electron microscopy revealed the developed bilosomal vesicular formulations to be spherical in shape. *In-vitro* release studies showed sustained release of drug from the developed formulations, compared with free drug suspension. *In-vivo* studies revealed enhancement of the pharmacological effect of rutin. The results revealed that the selected formulation, F2, ameliorated kidney dysfunction, oxidative stress and inflammation. In addition, it enhanced Akt/PI3K activation against PDC-induced AKI. This was confirmed by histopathology study. Accordingly, we can conclude that bilosomes could be a potential drug delivery system for enhancing the oral application and pharmacological activity of rutin.

## Supplementary Information

Supplementary Information.

## Data Availability

All data supporting the findings of this study are available within the paper and its Supplementary Information.
