# Supplementary material for: Rutin loaded bilosomes for enhancing the oral activity and nephroprotective effects of rutin in potassium dichromate induced acute nephrotoxicity in rats
Source: Sci Rep. 2024 Oct 11;14:23799. doi: 10.1038/s41598-024-73567-6 (PMC11479598; doi:10.1038/s41598-024-73567-6)
Supplement: Supplementary file 1 — Supplementary Information. [file 41598_2024_73567_MOESM1_ESM.docx]

|  |  |
| --- | --- |
| Supplementary Figure 1 | Supplementary Figure 2 |

**Supplementary Figure 1.** **TEM micrograph of selected rutin-loaded bilosomes F2.**

**Supplementary Figure 2. TEM micrograph of selected rutin-loaded bilosomes F6.**
